# Cooking Up Indoor Air Pollution: Emissions from Natural Gas Stoves

**DOI:** 10.1289/ehp.122-A27

**Published:** 2014-01-01

**Authors:** Wendee Nicole

**Affiliations:** Wendee Nicole was awarded the inaugural Mongabay Prize for Environmental Reporting in 2013. She writes for *Discover*, *Scientific American*, *National Wildlife*, and other magazines.

Natural gas cooking appliances, which are used by a third of U.S. households, can contribute to poor indoor air quality, especially when used without an exhaust hood.^1^ Gas stoves emit nitrogen dioxide (NO_2_), carbon monoxide (CO), and formaldehyde (HCHO), each of which can exacerbate various respiratory and other health ailments.^2^^,^^3^^,^^4^ In a study reported in this issue of *EHP*, researchers from Lawrence Berkeley National Laboratory and Stanford University developed a simulation model to estimate gas stoves emissions and the exposures experienced by different household members.^5^

The model used a sample cohort representing Southern California households, of which more than half use natural gas to cook. The investigators obtained data on the homes and the occupants, including how often they cooked breakfast, lunch, and dinner. The team estimated air exchange rate (i.e., the rate at which outdoor air replaces indoor air), the amount of time people spent at home, and outdoor profiles for NO_2_ and CO (indoor concentrations of these two pollutants are heavily influenced by outdoor levels, whereas HCHO concentrations typically depend on a variety of sources). They assumed one adult cooked in each home and that any children aged 0–5 years would be in close proximity to the adult while he or she was cooking.

Gas burners were estimated to add 25–33% to the week-averaged indoor NO_2_ concentrations during summer and 35–39% in winter. The variability between seasons likely reflected the fact that air ventilation is lower in winter. For CO, gas stoves were estimated to contribute 30% and 21% to the indoor air concentration in summer and winter, respectively. In this case, the appliances contributed relatively more CO during summer because outdoor concentrations tend to be lower then. The appliances added little to indoor HCHO concentrations relative to other indoor sources such as furniture and building materials.^5^

**Figure:**
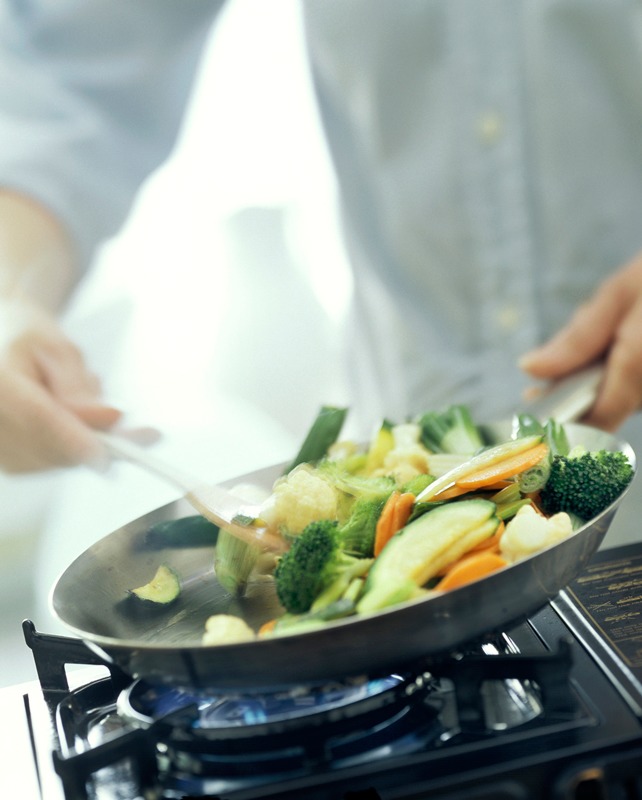
Emissions from gas stove burners can reach potentially harmful levels if the cook does not use a venting hood. © Food Photography by Eising/Corbis

The model predicted that when homes did not use venting range hoods, household exposures frequently exceeded benchmarks the authors set based on federal and state health-based standards.^6^^,^^7^ It also indicated that cooks and young children, who were assumed to be in closest proximity to the stove, would have the highest exposures.

Based on these modeling results, the investigators estimated that, during a typical winter week, 1.7 million Californians could be exposed to CO levels that exceed standards for ambient air, and 12 million could be exposed to excessive NO_2_ levels, if they do not use venting range hoods during cooking. “Clearly we have unhealthy situations indoors since we exceed outdoor standards in homes,” says lead author Jennifer Logue of Lawrence Berkeley National Laboratory.

In colder climates, people may not want to use vents because they send warm indoor air outside. But the authors suggest that increasing the use of venting range hoods could reduce indoor air pollution as well as exposures to these chemicals. Even greater reductions could be achievable with improved hoods that capture pollutants more effectively, or quieter hoods that people are more likely to turn on.

“A vent is a solution but not the only solution,” says Greg Diette, a Johns Hopkins University professor of medicine, epidemiology, and environmental health sciences. “Another solution is to swap out the stove [for an electric model].” Diette has also tested a promising air cleaner that adsorbs gases.

Charles J. Wechsler, an adjunct professor with the Environmental and Occupational Health Sciences Institute, Rutgers University, points out that adsorbents have finite life spans, but it can be difficult to know when it’s time to change them. “An exhaust system that incorporates a heat exchanger might be more promising,” he says. “Such units are used in Scandinavia.” Heat exchangers reduce heat loss to the outdoors.

Logue points out that simply cooking food, even on electric burners, also emits pollutants, especially particulate matter and acrolein. “Just switching from gas to electric will not solve all your pollution issues with cooking,” she says.
